# Supplementary figures and images for: Effects of cardiac contractility modulation on autophagy and apoptosis of cardiac myocytes in rabbits with chronic heart failure
Source: PLoS One. 2024 Dec 19;19(12):e0306242. doi: 10.1371/journal.pone.0306242 (PMC11658494; doi:10.1371/journal.pone.0306242)

LC3 Sham HF CCM

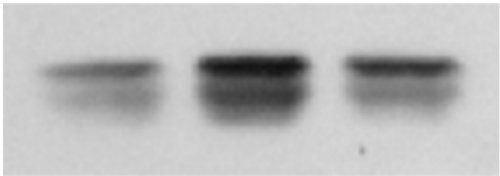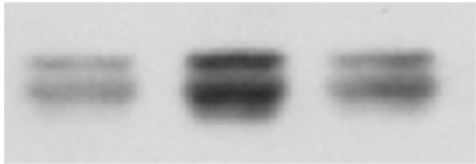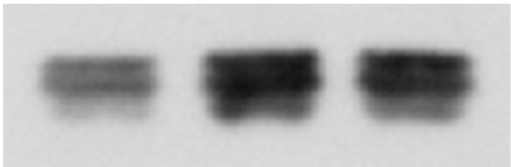

Beclin Sham HF CCM

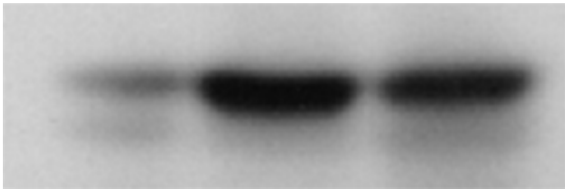

8463 38294 24193

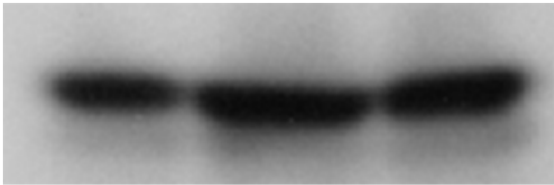

20483 42835 31029

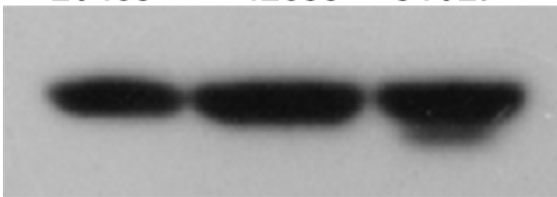

18643 39285 26783

P62 Sham HF CCM

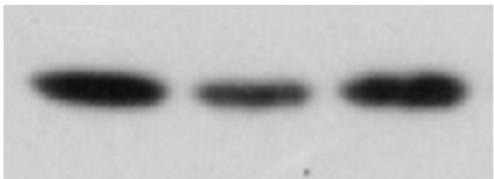

26473 7489 14693

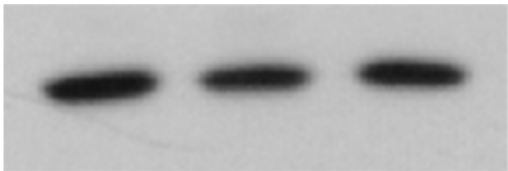

18946 5692 9182

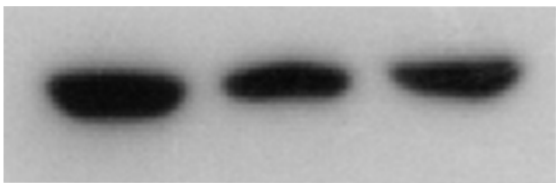

26452 12110 15683

Actin Sham HF CCM

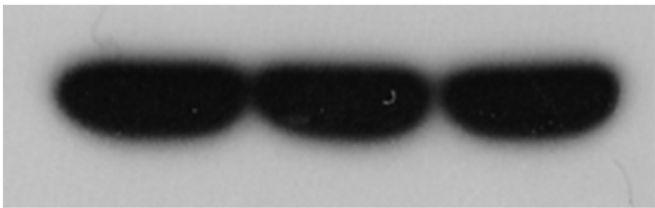

48736 46710 47822

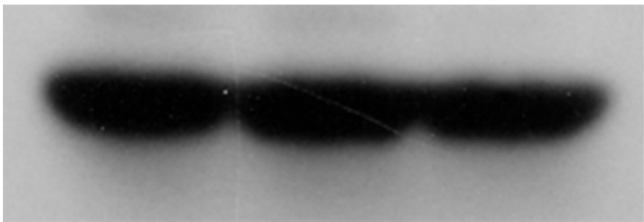

45362 46718 43287

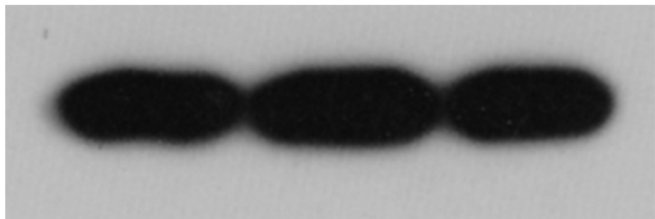

46372 48719 46892

Supplement: S1 Raw images — (PDF) [file pone.0306242.s002.pdf]
