# Supplementary material for: Effects of cardiac contractility modulation on autophagy and apoptosis of cardiac myocytes in rabbits with chronic heart failure
Source: PLoS One. 2024 Dec 19;19(12):e0306242. doi: 10.1371/journal.pone.0306242 (PMC11658494; doi:10.1371/journal.pone.0306242)
Supplement: Attachment — Submitted filename: Review Comments_1.pdf [file pone.0306242.s003.pdf]

Comments on manuscript PONE-D-23-21385, entitled **"Effects of cardiac contractility modulation on autophagy and apoptosis of cardiac myocytes in rabbits with chronic heart failure".**

There are overall formatting issues, line spaces are inconsistent. A native English speaker needs to be consulted for the language of the manuscript. Reports are required to be narrated in passive voice and not active voice. like 0.1g of the frozen tissue was processed for the analysis. The following are comments respective to the line numbers.
80- Requires checking for space between "C" and "for" at the end of the sentence.

81- It is referred not as "first" but primary antibody.

82- It is not "second" but secondary antibody.

83- Concentration of DAPI and the catalogue numbers with supplier details are missing.

84- Report what was the sealing agent and which microscope was used.

87- Report how much protein extract was used in the SDS-PAGE gel.

88- Statement needs restructuring- like "tissue extract was resolved by SDS-PAGE".

89- Sealing solution composition needs to be reported.

90- Catalogue and dilution of Primary and Secondary Antibody needs to be recorded.

91- "TTBS buffer" solution recipe needs to be added, Entire 2.3.2 needs revisions and elaborations.

96- Section 2.3.3 entire section needs revisions; change the voice.

97- Describe the procedure of RNA extraction briefly. Was any kit used, if yes state the manufacturer's name and catalogue. Add the sequences of the respective genes primers used.

105- Section 2.3.4 Procedure followed to do Flow Cytometry requires more details like what the probe was, etc. And needs a better analysis graph not only the raw images.

108- The catalogue number of the Roche kit for the TUNEL assay is required and briefly describe the process also.

111- Consistency in the Tense of English is required.

Fig 1 – Preferably add a quantification of the replicates for Immunofluorescence.

Fig 2a -Western Blot image looks squashed – fixation of dimensions required.

150- Spell check for Caspase

152 and Fig3a- Consistency is required in labelling one has LADH2 and the other has ALDH2 respectively. Has red underlines in the graph's labelling, so that needs to be fixed.

FIG 4, 5 – Requires proper data presentation form of graphs or in the form of four quadrants showing a comprehensive analysis between groups.

Fig 6 – Quantification of the images is required for a score.

Discussion- Unless the above-mentioned points are addressed, it is difficult to critique this section.
