# Supplementary material for: Effects of cardiac contractility modulation on autophagy and apoptosis of cardiac myocytes in rabbits with chronic heart failure
Source: PLoS One. 2024 Dec 19;19(12):e0306242. doi: 10.1371/journal.pone.0306242 (PMC11658494; doi:10.1371/journal.pone.0306242)
Supplement: Attachment — Submitted filename: Comments to rebuttal _ 7.pdf [file pone.0306242.s005.pdf]

Comments to rebuttal of the manuscript “Effects of cardiac contractility modulation on autophagy and apoptosis of cardiac myocytes in rabbits with chronic heart failure”

The revised manuscript has addressed the critical points made by reviewers. Addition of the statements regarding ethics statement is commendable. However, there persists some formatting issues, particularly in the graphical representation. In terms of language of communication, it also would require some minor corrections with the punctuations and the voice of the sentences. The following are the minor changes that needs to be completed before a final acceptance, according to me.

1. In Section 2.6, line 121, starting with “Carry out,….” needs to be changed to passive voice. And please insert the primer sequences of all the genes used in the study in a tabular form.

2. In Section 2.7, is the cell number 1X10^{5}cells/well or 1X105 cells/ml. Please address.

3. In Section 2.8, line 140, please change the term “baked” to “heated” as it is better alignment with the scientific reporting language. In line 142, “working liquid” can be omitted and replaced with the final concentration of the enzyme used for the experiment.

4. The line 164 needs to end in a full stop (.)and not colon (;). Similarly check other lines for punctuation errors.

5. For Fig1a, please see if a lower exposure image is available for both beclin and actin. The images at present are overexposed.

6. It is suggested that the authors follow an overall consistency in positionining of heading, astericks (*) or pound (#) in the graph as it is a standard and global practice used in scientific research. Also, if in the labelling of graph like “Sham group” is longer than to fitted in graph, either the authors reduce the font size or split in two lines. Otherwise, it is both difficult to comprehend and seems overlapping.

7. In section 3.4, Caspase-3 is mis-spelt.

8. For fig4-6, it is advised to add a bar graph to show the comparison between the groups in the study so that it is a better representation and comprehensive. For now, it seems to be more raw data images.
