# Supplementary material for: Effects of cardiac contractility modulation on autophagy and apoptosis of cardiac myocytes in rabbits with chronic heart failure
Source: PLoS One. 2024 Dec 19;19(12):e0306242. doi: 10.1371/journal.pone.0306242 (PMC11658494; doi:10.1371/journal.pone.0306242)
Supplement: Attachment — Submitted filename: Response to Reviewers.docx [file pone.0306242.s004.docx]

1、Requires checking for space between "C"and "for"at the end of the sentence.

37℃ for

2、It is referred not as "first"but primary antibody.

Change the first antibody to the primary antibody

3、It is not"second"but secondary antibody.

Change the second antibody to the secondary antibody

4、Concentration of DAPl and the catalogue numbers with supplier details are missing.

DAPI staining solution: Hebei Bohai Biological Engineering Company

5、Report what was the sealing agent and which microscope was used

sealing agent: Goat serum sealer

fluorescence microscope: Image-Pro Plus produced by American Media Cybernetics, Inc

6、Statement needs restructuring-like "tissue extract was resolved by SDS-PAGE".

According to the standard of 30μg protein per sample hole, the sample amount of each lane was calculated, and the sample was added to the prepared SDS-PAGE electrophoresis gel.

7、Sealing solution composition needs to be reported.

The sealing solution was prepared by adding 2.5g of 5% calf serum to TBST.

8、Catalogue and dilution of Primary and Secondary Antibody needs to be recorded.

the primary antibody was diluted by sealing solution: Beclin1 (1:1000), P62 (1:1000), LC3B (1:1000).

the secondary antibody was diluted by TTBS, Beclin1 (1:2000), P62 (1:2000), LC3B (1:2000).

9、“TTBS buffer”solution recipe needs to be added,Entire 2.3.2 needs revisions and elaborations.96-Section 2.3.3 entire section needs revisions;change the voice.

The frozen myocardium of 0.1g was weighed, split and centrifuged, and The frozen myocardium of 0.1g was weighed, split and centrifuged, and its protein content was determined by BCA method. According to the standard of 30μg protein per sample hole, the sample amount of each lane was calculated, and the sample was added to the prepared SDS-PAGE electrophoresis gel. The pre-dyed marker was added to the second lane on the left, and the loading buffer was added to the empty lane. The sealing solution was prepared by adding 2.5g of 5% calf serum to TBST. Through SDS-PAGE electrophoresis, the protein on the gel was transferred to the PVDF film and immersed in calf serum sealing solution. At 37℃, it was horizontally shaken and sealed for 1h. The primary antibody was diluted by sealing solution: Beclin1 (1:1000), P62 (1:1000), LC3B (1:1000). The closed PVDF membrane was removed and the TTBS was washed three times. PVDF membrane was transferred to the reaction box, appropriate amount of primary antibody was added, 4℃, horizontal oscillation, incubated overnight. Take out the PVDF membrane treated with primary antibody, rewarm it to room temperature naturally, and rinse it with TTBS oscillating for 3 times, 15min each time. PVDF membrane was transferred to the reaction box, secondary antibody was added (secondary antibody was diluted by TTBS, Beclin1 (1:2000), P62 (1:2000), LC3B (1:2000). At 37℃, it was horizontally shaken and incubated for 2h. After that, the film was washed with TTBS at room temperature. The luminous solution was dropped on the PVDF film, observed the film with an imager and photographed strips. ProPlus5.1 software was used for scanning and analyzing the gray value of the strip, and the relative expression level of target protein was expressed by the gray ratio of target strip to β-action strip.

10、Describe the procedure of RNA extraction briefly.Was any kit used,if yes state the manufacturer's nameand catalogue.Add the sequences of the respective genes primers used.

The 0.1g frozen myocardial tissue was weighed, ground and centrifuged, and the purity and integrity of RNA were detected by 1% agarose gel electrophoresis and type 756 ultraviolet spectrophotometer, it was reverse-transcribeed at 42℃ for 50min and reverse transcriptase was inactivated at 95℃ for 5 min. Carry out the PCR thermal cycle: 96℃, 4 min, and then three steps of reaction: 94℃, 30 s, 58℃, 30 s, 72℃, 30 s. Fluorescence signals were collected at the third step of each cycle and detected by Syber Green fluorescent quantitative PCR . (The fluorescence quantitative RT-PCR kit was made from Promega Company, USA）After amplification, GAPDH (The upstream primer: 5′ -TGAACGGGAAGCTCACTGG-3′, the downstream primer: 3′ -GCTTCACCACCTTCTTGATGTC-3′, and the amplified fragment length is 120 bp) was used as the internal reference gene for result analysis. Compared with the control group, the relative quantitative value (RQ value) of target gene expression was obtained, and the RQ value was used for statistical analysis.

11、Section 2.3.4 Procedure followed to do Flow Cytometry requires more details like what the probe was, etc.And needs a better analysis graph not only the raw images.

The cells of each group were inoculated into 6-well plates at a density of 1×105 cells/well and cultured for 48 h. The cells are collected after trypsin digestion. After 3 times of washing with PBS, the cells were re-suspended in 100 μL binding buffer and mixed with Annexin V-FITC (5 μL) and PI (10 μL) dye solution, dying at room temperature and away from light for 15 min, the apoptosis of cardiomyocytes was detected with Annexin V-FITC/PI apoptosis detection kit . Flow cytometry produced by BD Biosciences in the United States was used to detect according to the operating instructions.

12、The catalogue number of the Roche kit for the TUNEL assay is required and briefly describe the processalso.

The myocardium tissue was cut, dehydrated and immersed in wax, frozen and buried, and then cut into 3μm slices, then spread the slices in water bath and scoop the slices. The slices were baked for 1h in a constant temperature oven at 65℃, soaked in xylene for 30min, dehydrated with gradient alcohol and rinsed with tap water for 10min. The protease K working liquid was incubated at 37℃ for 15min, and 50μL Tunel reaction mixture was added to the sample, and the slices were placed in a humidor and incubated at 37℃ for 1h. 50μL converted POD was added and incubated at 37℃ for 30min. 50μL DAB substrate was added and incubated at room temperature for 10min. Hematoxylin was re-dyed, dehydrated, transparent and photographed. The Tunel kit produced by Roche was used to detect following the operating instructions. The apoptotic bodies were observed by optical microscope and the images were preserved.
