# Supplementary material for: Effects of cardiac contractility modulation on autophagy and apoptosis of cardiac myocytes in rabbits with chronic heart failure
Source: PLoS One. 2024 Dec 19;19(12):e0306242. doi: 10.1371/journal.pone.0306242 (PMC11658494; doi:10.1371/journal.pone.0306242)
Supplement: Attachment — Submitted filename: Point-to-point explaination.docx [file pone.0306242.s006.docx]

**Thanks for all your revisions, we will reply to them one by one, please point out again if there is any lack of details. Thanks again!!**

**1. In Section 2.6, line 121, starting with “Carry out,….” needs to be changed to passive voice. And please insert the primer sequences of all the genes used in the study in a tabular form.**

In Section 2.6, line 121, “Carry out,….” has been changed to passive voice. And we also insert a tabular form of the primer sequences of the genes used in the RT-PCR.

**2. In Section 2.7, is the cell number 1X10⁵cells/well or 1X105 cells/ml. Please address.**

In Section 2.7, it is the cell number is 1X10⁵cells/well.

**3. In Section 2.8, line 140, please change the term “baked” to “heated” as it is better alignment with the scientific reporting language. In line 142, “working liquid” can be omitted and replaced with the final concentration of the enzyme used for the experiment.**

We have changed the “baked” to “heated”.

We replaced “The protease K working liquid ” with “the pepsin at 0.5% concentration”.

**4. The line 164 needs to end in a full stop (.)and not colon (;). Similarly check other lines for punctuation errors.**

Thanks for the advice,we did find some errors and made changes.

**5. For Fig1a, please see if a lower exposure image is available for both beclin and actin. The images at present are overexposed.**

The figure we got are indeed like this. In order to ensure that all the figures have a consistent format, we had to adjust the size of the figures. If you need the original figure, we have already provided it in the original data.

**6. It is suggested that the authors follow an overall consistency in positionining of heading, astericks (*) or pound (#) in the graph as it is a standard and global practice used in scientific research. Also, if in the labelling of graph like “Sham group” is longer than to fitted in graph, either the authors reduce the font size or split in two lines. Otherwise, it is both difficult to comprehend and seems overlapping.**

Revised

**7. In section 3.4, Caspase-3 is mis-spelt.**

Revised

**8. For fig4-6, it is advised to add a bar graph to show the comparison between the groups in the study so that it is a better representation and comprehensive. For now, it seems to be more raw data images.**

Actually,there are specific values for apoptosis rates in the image, but it is true that the bar chart is a better comparison of the differences between groups, which has been modified.

**1.Authors provided a very short description of the chronic heart failure (CHF) and also mention the current/previous treatment methods (mostly beta-blocker) that have been attempted to treat this disease. However, for the general reader, it would be quite challenging to understand what is CHF and what happens during this phase. Moreover, it would be beneficial for the readers to briefly state the mode of action of the beta-blocker and how/why this method of treatment is less-effective (if it is). Why do the authors think that CCM can be a better strategy to treat CHF?**

After the first revision, we further improved the article and uploaded a new WORD named "Manuscript", in which the definition of heart failure and the current treatment methods were briefly introduced. However, β-blockers are not specifically discussed as part of current drug therapy. At present, heart failure is still a disease that needs to be explored, and our animal experiments provide evidence that CCM can help improve heart function in rabbits with heart failure, thereby treating chronic heart failure, therefore, we believe that CCM can improve the prognosis of patients with heart failure to a certain extent, and may have a better treatment effect together with other treatments, but these still need to be further discussed.

**2. In the line 45-47, authors used a term “molecular modeling”. What does it mean? Molecular modeling of what? How is it happening? (Sorry, if I missed that info somehow!).**
 This is a sentence from the first edition of the manuscript that describes the mechanism of action of CCM. **“CCM is a series of non-excitatory signals which can be applied during the absolute refractory period to enhance the strength of left ventricular contraction without increasing myocardial oxygen consumption^[7-8]^. The process of contraction force increasing is mediated by reversing the molecular remodeling and restoring the expression of several calcium-handling proteins in myocardial hypertrophy with heart failure ^[9]^.**” Heart failure (HF) involves changes in cardiac structure, myocardial composition, myocyte deformation, and multiple biochemical and molecular alterations that impact heart function and reserve capacity. Collectively, these changes have been referred to as 'cardiac remodeling'. “molecular modeling”there means it.

But we rewrote part of the article, which has now been removed from the new manuscript.

**3. The methods section is well-written. However, materials section requires more details about the reagents and instruments that were used in these experiments.**

We've added to this section.

**4. The line 79 starts with “The slices were stored...”. Slices of what?**

We've added content on heart processing in experimental animals. ”After 16 weeks post ascending aortic constriction (AAC) or sham surgery, the animals in each group were euthanized with an overdose of anesthesia. The hearts were excised and flushed with physiological saline to remove any residual blood from the heart chambers. The surface liquid of the hearts was blotted dry with filter paper, and the heart weights were recorded. Myocardial tissue from the anterior wall of the left ventricle was collected using ophthalmic scissors and fixed in 4% paraformaldehyde for one day for immunofluorescence staining. The tissue underwent routine dehydration with a graded alcohol series (70%, 80%, 90%, 100%), clearing with xylene (three times), wax infiltration at 65°C, embedding in paraffin blocks, and sectioning into 4μm consecutive slices.”

The slices of the myocardial tissue.

**5. In figure 3, the authors checked the expression level of Bcl-2, Bax, Caspase-3 and LADH2 in Sham, HF and CCM groups and concluded that the expression of these mRNAs and the respective proteins in cardiomyocytes can reverse the development of cardiomyocytes apoptosis. Can authors also check the protein levels (by western blotting) from those differently expressed mRNA in Sham, HF and CCM groups and validate the above-mentioned statement?**

Unfortunately, we did not further verify that the expression of these mRNAs and their proteins can reverse the development of cardiomyocyte apoptosis by measuring the level of protein expression in the experimental design, and this indicator can be further improved in future experiments.

**6. Section 3.6 (line 176-180), authors looked at the cardiomyocytes apoptosis level by TUNEL assay, where they found that overall the apoptosis rate in HF and CCM group was increased compared with that in Sham group. Do authors have any justification of this observation? Also, is there any way to quantify this data (including the statistical significance) to get a better analysis of the apoptosis level?**

We can determine the apoptosis rate of myocardial cells by observing the number of stained cells in TUNEL-stained slices. The article also statistically analyzed the specific apoptosis rate. The Sham group, as the normal rabbits in the control group of the experiment, should have the lowest apoptosis rate. However, CCM treatment has an improvement effect on the hearts of heart failure rabbits, so the apoptosis rate should be higher than the Sham group but lower than the HF group.

**7. Discussion requires more details and proper explanation of the results that authors presented in the manuscript. What is the molecular mechanism behind the effectiveness of CCM treatment in case of chronic heart failure? How does up regulation/down regulation of those proteins tested here in CCM group of animals justify this treatment mechanistically during chronic heart failure? In the discussion section, authors mentioned that previously they showed the positive effect of CCM in cardiac function, however, did not cite any of their publication. Why?**
We have rewritten the discussion section in the revised manuscript with the document named “Manuscript”.
